# A Model for Statistical Regularity Extraction from Dynamic Sounds

**DOI:** 10.3813/AAA.919279

**Published:** 2018-12-07

**Authors:** Benjamin Skerritt-Davis, Mounya Elhilali

**Affiliations:** Johns Hopkins University, Baltimore, Maryland, United States. mounya@jhu.edu

## Abstract

To understand our surroundings, we effortlessly parse our sound environment into sound sources, extracting invariant information—or regularities—over time to build an internal representation of the world around us. Previous experimental work has shown the brain is sensitive to many types of regularities in sound, but theoretical models that capture underlying principles of regularity tracking across diverse sequence structures have been few and far between. Existing efforts often focus on sound patterns rather the stochastic nature of sequences. In the current study, we employ a perceptual model for regularity extraction based on a Bayesian framework that posits the brain collects statistical information over time. We show this model can be used to simulate various results from the literature with stimuli exhibiting a wide range of predictability. This model can provide a useful tool for both interpreting existing experimental results under a unified model and providing predictions for new ones using more complex stimuli.

## 1. Introduction

Regularity extraction is an essential aspect of auditory object perception, in which the brain extracts useful information from sounds over time to interpret our surroundings [1]. This ability is often studied in the literature using deviance detection experiments, where listeners are presented with a sequence of sounds exhibiting some regularity and responses are compared between the members of the regularity and deviations from the regularity for signs of detection in the brain [2]. Behavioral and neural evidence has shown the brain is sensitive to a variety of regularities, with the mismatch negativity (MMN) as a typical marker of deviance detection in electroencephalography (EEG) and magnetoencephalography (MEG) [3].

The manner in which these regularities are represented in the brain is unknown. A repeating pattern could be represented explicitly as a “template”, but this mechanism would be computationally inefficient to represent the vast richness of natural sounds in the brain. It is more plausible that the brain employs a statistical description of sounds that incorporates uncertainty to robustly abstract out invariant information. Existing models are limited in scope and generalizability, either representing only repeating patterns [5] or computationally constrained to a small set of discrete symbols [6], rather than sounds that vary along a continuum.

We use a perceptual model that embodies Bayesian theories of perception, collecting statistical representations of sounds [7, 8]. To demonstrate its utility, we show this model accounts for many findings in the literature from the regularity extraction canon, re-casting existing results in terms of *statistical regularity extraction.*

## 2. Model Description

We use the Dynamic Regularity Extraction (D-REX) model^1^ presented in [9] to simulate findings in the literature. This model is based on a Bayesian inference framework designed to perform sequential predictions in dynamic sequences containing unknown changes in underlying statistical structure [10, 11].

The input to the model is a sequence of observations *{x*_*t*_*}* assumed to be distributed according to a probability distribution with unknown parameters *θ*; presently, observations are limited to a sequence of tone frequencies from a single sound source. The model sequentially builds a predictive distribution for the next observation at time *t +* 1 using sufficient statistics $\hat{\theta }$ collected over the observed sequence: $P\left(x_{t+1}|x_{1:t}\right)=P\left(x_{t+1}|\hat{\theta }\left(x_{1:t}\right)\right).$

The underlying distribution is assumed to be a D-variate Gaussian, where the dimensionality *D* specifies the amount of temporal covariance collected by the model; for example, a model with *D* = 1 collects marginal statistics (mean and variance), while a model with *D* = 3 additionally collects joint statistics (covariances) between *x*_*t*_*, x*_*t*-1_, and *x*_*t*-2_.

The model assumes the parameters *θ* change at un-known changepoint times. All following observations are then independent of those preceding the change, thus limiting the context window of observations relevant for the parameter estimates $\hat{\theta }.$ Because changepoints must be inferred from the observations, the model maintains multiple hypotheses across different contexts and then “integrates out” the context to build a prediction that adapts to un-known changes:

(1) $$P\left(x_{t+1}|x_{1:t}\right)=\sum_{c_{t}}P\left(x_{t+1}|c_{t},x_{t-c_{t}+1:t}\right)P\left(c_{t}|x_{1:t}\right).$$

In the sum, the first term is the prediction given the context *c*_*t*_ (which only depends on observations *within* the context window); this is weighted by the second term, the model belief that the current context *is c*_*t*_. With each incoming observation, the sufficient statistics for each context *c*_*t*_, as well as the beliefs, are updated incrementally (see [9] for details).

### 2.1. Perceptual parameters

As described thus far, the model makes Bayes-optimal predictions in the presence of changepoints [10]. To introduce more perceptual plausibility, we impose two constraints on the model. First, a memory parameter (***M***) represents finite working memory capacity, limiting how many past observations can be used to build predictions and, by extension, the number of context windows that can be maintained. Second, an observation noise parameter (***N***) sets a lower bound on prediction uncertainty. These parameters represent variabilities in perceptual abilities across individual listeners and allow for a range of behaviors from the model.

### 2.2. Surprisal response

With each observation, the model outputs prediction error, or *surprisal: $S_{t}=-\log P\left(x_{t+1}=X_{t+1}|x_{1:t}\right),$*where *X*_t+1_ is the observation at time *t +* 1. Note that an observation with low predictive probability has high surprisal and vice versa. In the Results section, we compare this surprisal response from the model to deviance responses in neural results from the literature.

## 3. Results

We collected surprisal responses from the D-REX model to stimuli found in the deviance and change detection literature. Stimuli range in predictability to show the capacity of the model to capture a variety of phenomena under a single framework. Using different sets of statistics in the model (via the dimensionality *D*), we can ascertain the statistics that are sufficient—the “simplest explanation”— for responses observed in the brain.

**Figure 1.**
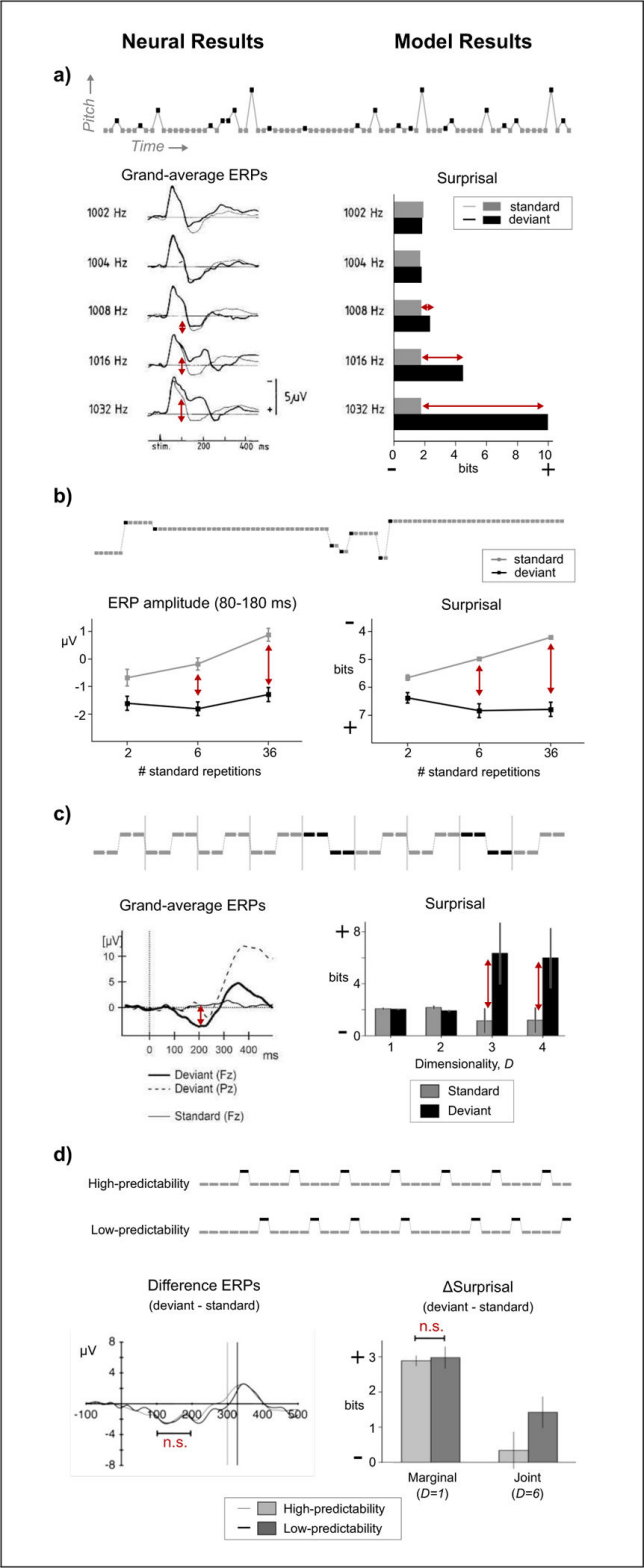
(Colour online) Neural results from the literature (left) are compared to surprisal responses from the D-REX model (right) to the same stimuli (above): a) [12], b) [13], c) [14], d)[15]. Arrows indicate replicated trends. Surprisal axis is occasionally inverted to facilitate visual comparison. Experimental figures reproduced with permission from the publishers. In b), experimental figure generated from Table 1 in [13].

**Figure 2.**
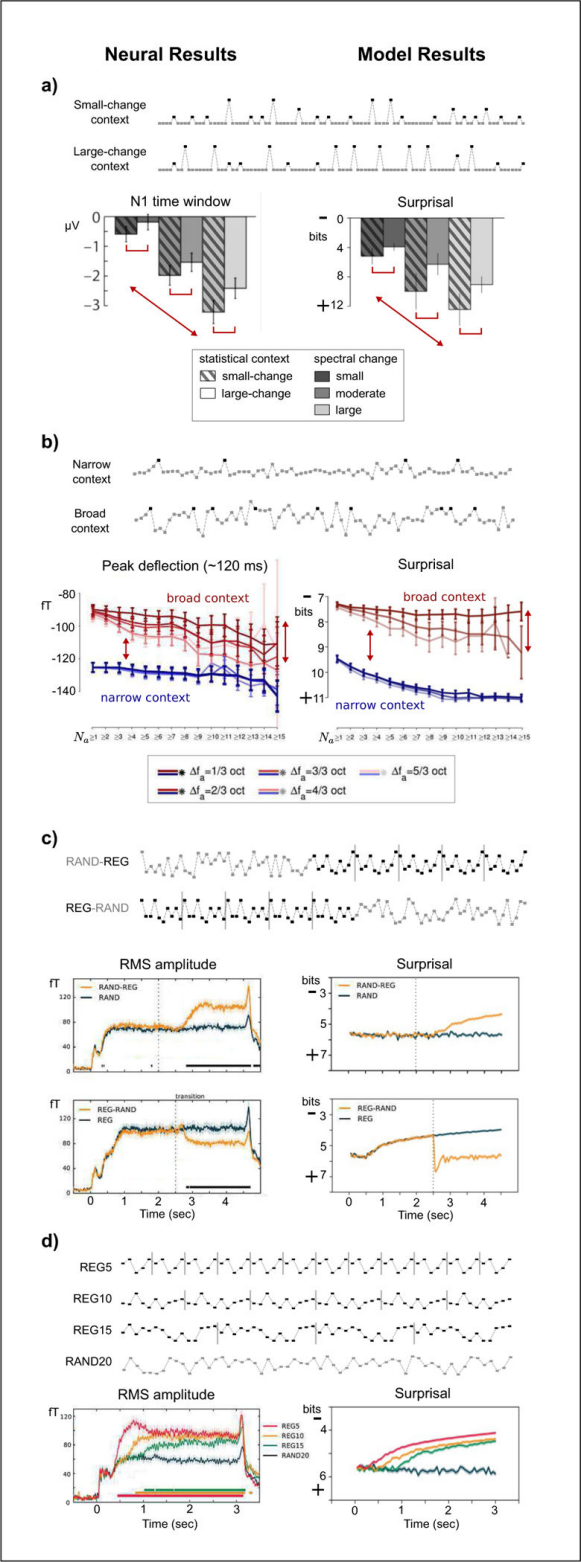
(Colour online) Comparison of neural and model results, continued. a) [16], b) [17], c) and d) [18]. Arrows indicate replicated effects. Surprisal axis is occasionally inverted to facilitate visual comparison. Experimental figures reproduced with permission from the publishers.

In Figures 1 and 2, neural results directly from the literature are presented alongside model results for comparison (e.g., MMN amplitude vs. surprisal), with example stimuli shown above each result. Trends shared between neural and model results are indicated by red arrows. To facilitate visual comparison, the surprisal axis is occasionally inverted to align higher surprisal in the model results with lower predictability in the neural results. Figures from the literature are reproduced in their original form, where possible.

### Oddball.

Dating back to 1978, Näätänen and colleagues have used the oddball paradigm to elicit neural markers of deviance from a detected regularity [3, 4]. The paradigm includes a *standard* stimulus exhibiting some regularity and *deviant* stimuli breaking the regularity; if the brain is sensitive to the regularity, the mismatch negativity (MMN) appears around 100–200 ms after onset in the deviant’s Event-Related Potential (ERP) response relative to the standard. This negativity increases with frequency distance between the deviant and standard [12]. The D-REX model with *D =* 1, or marginal statistics, similarly shows an increase in surprisal to the deviant as frequency distance increases (see Figure 1a).

### Roving oddball.

The oddball paradigm has been extended using a standard that changes over time, where each deviant becomes the new standard. As the number of standards increases, ERP response to the *standard* increases in the MMN window (80–180 ms), while response to the *deviant* stay relatively the same [13]; similarly, as the number of standards increases, model surprisal with *D* = 1 decreases (*F*_2_,_147_ = 108.1,*p* < 0.0001), while surprisal to deviants stays the same (*F*_2_,_147_ = 1.18,*p* > 0.1) (see Figure 1b, surprisal axis flipped for visual comparison).

### Pattern oddball.

Tone-patterns can also serve as standards in the oddball paradigm. In [14], an MMN response to the first tone of the deviant pattern (BBAA) relative to the first tone of the standard pattern (AABB) indicates the brain is sensitive to the 4-tone pattern. In the model’s surprisal response, this is replicated with dimensionality *D >* 2 (*t*_74_ = 15.1,*p* < 0.0001), indicating the minimal statistics necessary to detect the deviant is actually over a shorter window than the pattern itself; deviance can be detected by the entire 4-tone pattern or by three repetitions of the same tone (see Figure 1c).

### High- & low-predictability oddball.

Top-down attentional affects have been measured in the MMN response. In [15], the MMN response was measured in two conditions: a high-predictability condition where the number of standards preceding a deviant was usually 4 (AAAAB), and a low-predictability condition where the number of standards was uniformly distributed between 2 and 6. Listeners were tasked with detecting every deviant (B). ERP evidence shows a significant MMN response to deviants but *no difference* in MMN magnitude between predictability conditions; this null result is replicated by differential surprisal betweeen deviant and standard from the model with *D* = 1 collecting only marginal statistics (*t*_23_ = 1.27*,p >* 0.1) (see Figure 1d).

By contrast, a model with *D* = 6 collects temporal covariances that cover the entire AAAAB pattern and no longer finds the final B tone “surprising” (see Figure 1d-right). This mirrors a similar study where listeners were tasked with listening for the entire pattern and exhibited no MMN response to the deviant tone [19]. These top-down effects can be described in terms of the statistics being collected—when attending to the B tone only, listeners collect marginal statistics; when attending to the entire AAAAB pattern, listeners collect long-range temporal statistics.

### Statistical oddball biased toward large or small changes.

Context effects have been observed in the MMN response by manipulating the relative probabilities of deviants, biasing them toward small- or large-change deviants [16]. Effects due to spectral change (between deviant and standard) and statistical context are observed in N1 amplitude: magnitude increases with spectral change and is augmented by the small-change context, where large changes are less probable. An ANOVA applied to model surprisal (with *D* = 1) shows the same significant effects for spectral change (*F*_2_,_477_ = 668.66,*p* < 0.0001) and statistical context (*F*_1,477_ = 221.14,*p* < 0.0001) (see Figure 2a).

### Gaussian sequences differing in variance.

Context effects have also been observed using random stimuli drawn from a Gaussian distribution with different variances [17]. Responses to deviants (presented 2 octaves above the mean) show a negative peak around 120 ms that is larger for narrow relative to broad statistical context. Additionally, there is evidence of adaptation effects in the broad context when comparing deviant responses based on the number of preceding tones *(N*_*a*_*)* falling outside a fre- quencyregion (Δ*F*_*a*_) (see [17] for details). The model with *D* = 1 replicates these results (see Figure 2b).

### Regular vs. random sequences.

Repeating patterns are another class of stimuli used to explore regularity extraction in the brain. In particular, RMS power in MEG has been shown to increase with decreasing entropy in the stimulus [18]: RMS power *increases gradually* when the stimulus transitions from random to repeating pattern (RAND-REG), while RMS power *decreases abruptly* for the opposite transition (REG-RAND). The model replicates both of these phenomena in the time-course of sur- prisal, with *D* greater than the pattern length (see Figure 2c). Additionally, the model replicates effects of pattern length on RMS power [18], again reflecting differences in entropy (see Figure 2d).

## 4. Conclusion

The D-REX model utilizes statistical descriptions of sound sequences to replicate findings across a wide swath of the regularity extraction literature. While these statistical descriptions may not be *necessary* to replicate these findings, the model provides a unified interpretation that is more generalizable toward natural sounds, where randomness and noise abound. Beyond retrospective intepreta-tion of existing results, the D-REX model can be used to guide future experiments probing the temporal processing of more complex sounds. Moreover, since the model employs a Bayesian framework that is agnostic to probability distributions and underlying statistics, the D-REX model offers a generalized platform to explore sufficient statistics underlying regularity tracking in the auditory system, as well as contrast different interpretations of behavioral and neurophysiological results for parsing complex sound sequences.

## Footnotes

1 code available at https://engineering.jhu.edu/lcap/software
